# Analysis of vascularization in thyroid gland nodes with superb microvascular imaging (SMI) and CD34 expression histology: a pilot study

**DOI:** 10.1186/s12880-021-00690-5

**Published:** 2021-10-30

**Authors:** Thomas Studeny, Wolfgang Kratzer, Julian Schmidberger, Tilmann Graeter, Thomas F. E. Barth, Andreas Hillenbrand

**Affiliations:** 1 Department of Internal Medicine I, Ulm University Hospital, Albert-Einstein-Allee 23, 89081 Ulm, Germany; 2 Department of Diagnostic and Interventional Radiology, Ulm University Hospital, Albert-Einstein-Alee 23, 89081 Ulm, Germany; 3 Institute of Pathology, Ulm University Hospital, Albert-Einstein-Allee 23, 89081 Ulm, Germany; 4 Department of General and Visceral Surgery, Ulm University Hospital, Albert-Einstein-Alee 23, 89081 Ulm, Germany

**Keywords:** Ultrasound, Thyroid, Vascularisation, Cancer, Superb microvascular imaging

## Abstract

**Background:**

The Doppler sonography technique known as "superb microvascular imaging" (SMI) is advancing sonographic micro vascularization imaging in various disciplines. In this study, we aimed to determine whether SMI could reliably reproduce the blood flow in thyroid nodes and whether malignancy could be diagnosed, based on vascularization properties. Immunhistochemical staining by CD34 and SMI where used to determine the vascularization of nodes in terms of quantified vascularization parameters gained by computational evaluation.

**Methods:**

We used image analysis programs to investigate whether the quantitative value for vascularization strength in the thyroid node, measured with SMI, was correlated with the actual degree of vascularization, determined microscopically. We included 16 patients that underwent thyroid resections. We prepared thyroid gland tissue slices for immunohistochemistry and labelled endothelial cells with CD34 to visualize blood vessels microscopically. We used image analysis programs, ImageJ, to quantify SMI Doppler sonographic measurements and CellProfiler to quantify CD34 expression in histological sections. We evaluated the numeric values for diagnostic value in node differentiation. Furthermore, we compared these values to check for correlations.

**Results:**

Among the 16 nodes studied, three harboured malignant tumours (18.75%): two papillary and one follicular carcinoma. Among the 13 benign lesions (81.25%), four harboured follicular adenomas. Malignant and benign nodes were not significantly different in sonographic (0.88 ± 0.89 vs. 1.13 ± 0.19; *p* = 0.2790) or immunohistochemical measurements of vascularization strength (0.05 ± 0.05 vs. 0.08 ± 0.06; *p* = 0.2260).

**Conclusion:**

We found a positive, significant correlation (r = 0.55588; *p* = 0.0254) between SMI (quantitative values for vascularization strength) and immunohistochemistry (CD34 staining) evaluations of thyroid nodes.

## Background

About half the population will develop thyroid nodules during their lifetime [1–3]. Thyroid nodules are typically discovered randomly, during a physical or ultrasound examination [4, 5]. In a few cases (5–10%), the nodules are caused by a malignant tumour, and of these, about 60% are caused by papillary carcinoma [6].

Ultrasonography is a powerful method for assessing the risk of malignancy in a thyroid node [7–9]. Classification systems based on grayscale imaging criteria, such as the Thyroid Image Reporting and Data System, have been established to assess malignancies. Similarly, elastography was proven to be a useful diagnostic tool [8, 10–12]. However, the diagnostic significance of classical Doppler sonography techniques, such as colour Doppler and Power Doppler, remains controversial. It is uncertain whether hypervascularization or certain types of blood circulation indicate malignancy in a node [13–16]. This controversy is reflected in the different established diagnostic guidelines used by different endocrinological societies [17, 18]. For example, intranodular hypervascularization is not part of the diagnostic scheme recommended by the American Thyroid Association, but in the British Thyroid Association, this blood circulation pattern is considered a criterion for malignancy.

It was previously shown that “superb microvascular imaging” (SMI) provided a more precise depiction of the blood flow in thyroid nodes; thus, imaging microvascular flow could be significantly improved [6, 19–23]. In some cases, vascularization types that could not be determined with colour Doppler and Power Doppler could be determined with SMI. SMI is a Doppler sonographic method that measures microvascular blood flow. It uses an intelligent algorithm to suppress flow and motion artefacts, and thus, it provides highly sensitive detection of small blood vessels with low flow velocities. In monochrome SMI mode, the strength of the flow signal is represented as a corresponding gray intensity value per pixel unit; alternatively, in colour-coded SMI mode the flow signal strength is represented with different colours. Previous studies have shown that monochrome SMI provided more vascular and flow imaging detail and had higher sensitivity than colour-coded SMI [2, 24]. Therefore, in the present study, we used the monochrome SMI Doppler sonographic method to evaluate node perfusion.

New vessel formation and angiogenesis are pathogonomic for neoplasia and obligatory for tumour progression [25]. Several studies have shown that tumour tissues expressed elevated levels of angiogenic and lymphogenic factors, such as VEGF, KDR, COX-2, p27, CD31, and CD34 [26–28]. Most thyroid malignancies are characterized by a high fraction of microvessels that are < 100 µm in diameter; however, this size is below the detection sensitivity of conventional Doppler techniques [26]. Thus, SMI might prove useful for the detection of microvascularization, similar to the usefulness of sonography for detecting breast node, liver, or testicular cancers [29–32]. We hypothesized that the strength of vascularization in the ultrasound image correlates with the expression of angiogenic factors. We compared the strength of vascularization in the thyroid gland node, determined with SMI, to the degree of vascularization observed microscopically on CD34-labelled sections. We used CD34, because it is a specific marker for all vascular endothelial cells. The image analysis programs, ImageJ and CellProfiler, were used to evaluate, quantify, and compare Doppler sonographic measurements to histological results. Furthermore, we investigated whether the disposition and nodule blood supply between benign and malignant nodules could be judged, based on SMI methods.

## Methods

### Patient collective

This prospective pilot study recruited patients with operable thyroid findings that had been referred to the Department of Visceral Surgery at Ulm University Hospital. All patients were informed orally and in writing about the study, and all provided written consent. We included patients with a thyroid gland node that were scheduled for a thyroid (partial) resection. We excluded patients that had basic thyroid diseases, such as Hashimoto or Graves' disease, or previous tissue manipulations, such as an fine needle aspiration or partial resection. All patients were examined with ultrasound the day before the planned thyroid operation. The ultrasound was performed by a very experienced investigator with more than five thousand examinations per year. In cases of multiple nodes, the most conspicuous resp. suspicious node per thyroid lobe or isthmus was used in the examination. Hereby conspiciousness resp. suspiciousness was assessed subjectively by researcher.

### SMI examination and computer-aided evaluation

Thyroid blood flow behavior was examined in monochrome SMI mode. Blood flow was quantified in the obtained images using the open-source image processing program ImageJ, which determines the vascularization quotient (VQ). The quotient is obtained from the ratio of thyroid lobes adjacent to the nodule that corresponded to the ROI. Our presented determination of the quotient is comparable to the determination of the so called vascularization index (VI) in SMI mode, which is integrated as a software tool in high-end Toshiba/Canon ultrasound systems.

**Fig. 1:**
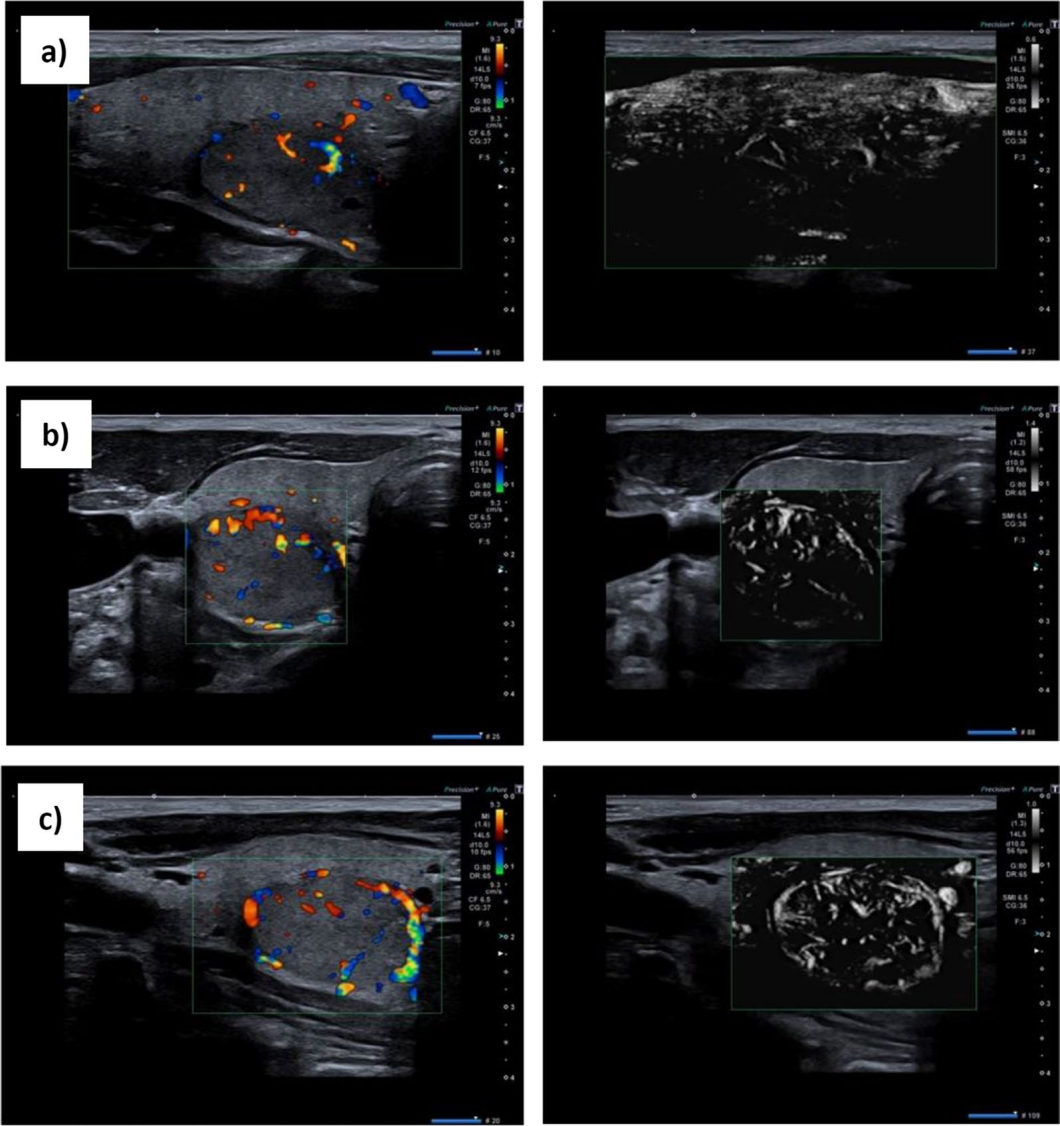
User interface of a Toshiba Aplio i800 ultrasound imaging system. (Left panels) Doppler sonogram displayed in colour Doppler mode; (right panels) the same sonogram displayed in monochromatic SMI mode. **a** Creation of the ROI total (green rectangle); **b** creation of the ROI-A (green square) in the cross-sectional view and **c** ROI-A (green rectangle) in the longitudinal view

All sonographic examinations were performed with the ultrasound device, Aplio i800 (Toshiba/Canon), equipped with software version V2.3. We used the ultrasound probe, 14L5, at 10 MHz. At the beginning of the examination, the thyroid gland was imaged with the default settings implemented by the manufacturer. When the ultrasound device was switched to colour Doppler mode, the same settings were retained, and a so-called region of interest (ROI) was defined by the examiner. Within the ROI, the Doppler sonographic information was displayed graphically. When switching from colour Doppler to SMI mode, the default settings and the ROI size and position were retained from the colour Doppler mode. In SMI mode, an image sequence was created for the total ROI, which represented the complete thyroid lobe. The node was displayed in a longitudinal section, and this area was called *ROI total* (Fig. 1a). Furthermore, within the *ROI total*, several ROI-As were defined, which contained the complete node in the longitudinal and cross-sectional planes, along with parts of the surrounding thyroid tissue (Fig. 1b, c). Two images were acquired in each plane of the section. Additionally, in SMI mode, a ROI-B was selected that showed the blood flow at a point in the thyroid lobe adjacent to the node that corresponded to the ROI-A. When possible, the ROI-B was the same size and located at the same depth as the ROI-A.

**Fig. 2:**
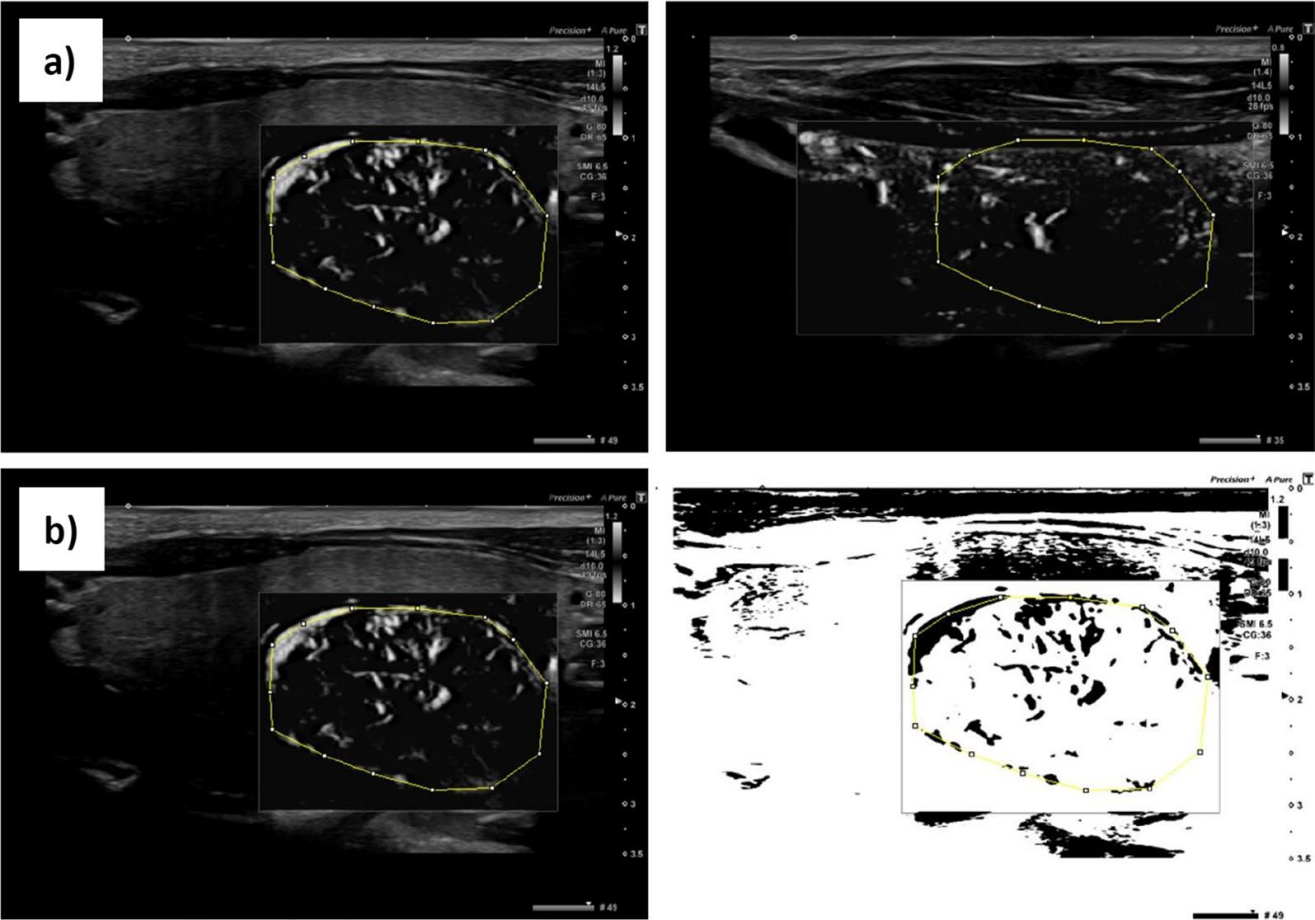
Determination of quantitative vascularization parameters in SMI mode. Images show the workings of the image processing software, ImageJ. **a** In the SMI sonogram window, an area is selected (yellow polygon) to highlight nodes and parenchyma. The selected areas are listed in the ROI Manager window, and the grayscale mean values are determined. **b** For the selected node (yellow polygon) in the SMI sonogram image, a threshold is set, and areas are extracted that have grayscale pixel values above the threshold. The result is an image sequence that only contains areas with a positive flow signal. The measured value is the fraction of these areas within the total selected area of the node

The image analysis program, ImageJ, provides a gray-value analysis for quantitative evaluations of monochrome SMIs. This mode has been used previously to address other questions [33, 34]. In the present study, we used ImageJ version 1.51J8 to quantify vascularization in the ROI-A and ROI-B, in both longitudinal and cross-sectional views. In the longitudinal view, we measured the average gray value of an area in the ROI-A (*ROI longitudinal*; Fig. 2a). In addition, we determined the fraction of area that contained pixels with a positive flux within the total ROI-A area (*ROI longitudinal area fraction*, Fig. 2b). Then, we measured the same parameters in the cross-sectional view to obtain the *ROI cross* and *ROI cross area fraction*, respectively. Finally, we divided each of these parameters measured in ROI-A images by their equivalent parameters measured in the ROI-B images to obtain the parameters *Quotient longitudinal*, *Quotient longitudinal area fraction, Quotient cross*, and *Quotient cross area fraction*. We also obtained the *Quotient all* and *Quotient all area fraction* by dividing the quantified vascularization values (average gray value and fraction of area that contained pixels with a positive flux) measured in the nodal area of *ROI total* by its counterpart in the parenchymal area, near the node in the same ROI.

### Immunohistochemical examination

After the patient had undergone surgery, the resected thyroid gland was sent to the clinic's internal Surgical Pathology Department for node differentiation and histochemistry. A distinction was made between benign (struma node, parenchymal nodes, micro-, normo- or macrofollicular adenomas, and cysts) and malignant entities (papillary, follicular, medullary, and undifferentiated carcinomas). After formalin fixation and paraffin embedding, thin tissue sections were prepared with the Microm HM 450 for light microscope examinations. These sections were stained with haematoxylin–eosin. The thyroid gland sections were also stained immunohistochemically. Briefly, the tissue sections were de-waxed with xylene and alcohol. Ethyldiaminetetraacetic acid, pH 9.0, completely reduced sulphur, pH 6.1, and finally, Ethyldiaminetetraacetic acid pH 6.1 were used for pretreatment with a steamer. After incubation and rinsing with distilled water, the sections were stained with anti-CD34 (DAKO) as the primary antibody. The sections were then rinsed with a phosphate-buffered saline solution. In the next step, the preparations were incubated with a biotinylated secondary antibody, streptavidin, RED, and haemalaun for 16 min, according to the ABC method. Sections were rinsed with the buffer after each staining step. Finally, the incisions (sections) were covered with Aquatex.

### CD34 and computer-aided analysis

As a reference that represented the actual strength of the blood flow in the thyroid node, we determined the vascularization strength in CD34-stained thyroid sections microscopically. This value was quantified with the image processing programs, CellProfiler and ImageJ. This reference value was compared to the blood flow measured quantitatively with SMI and ImageJ analyses.

**Fig. 3:**
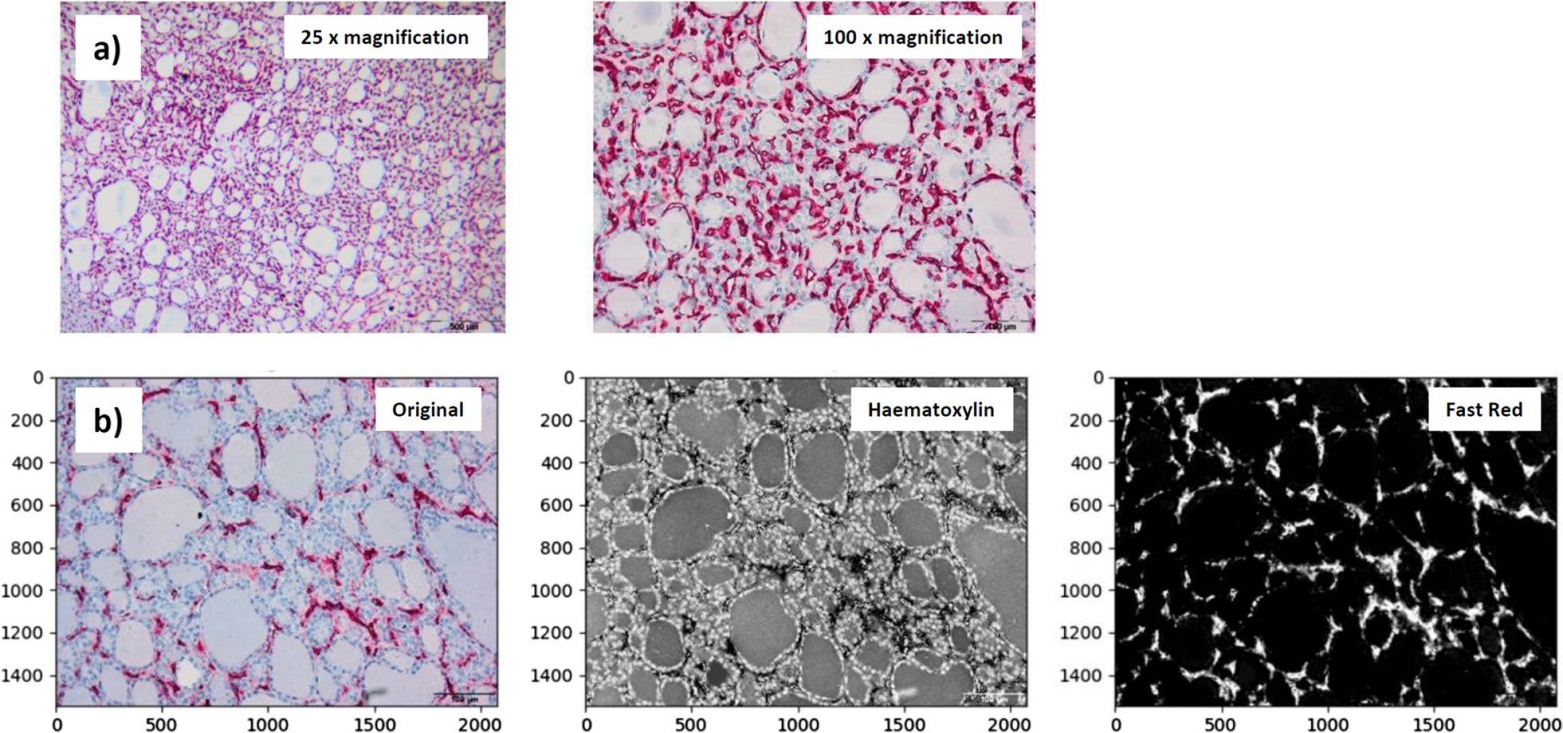
Microscopic images of a CD34-labelled immunohistological tissue section of a thyroid node. **a** The node is shown at (left) 25× and (right) at 100× magnifications. The pink colour corresponds to CD34-labelled areas. During image acquisition, the camera and microscope settings were adjusted to ensure that the colour had a similar tone for all samples. **b** Demonstration of the UnmixColours module: Image sequences are shown for (left) the original image file, (centre) the extracted image of exclusively haematoxylin-stained content, and (right) the extracted image of exclusively fast-red-stained areas. In the centre and right images, white represents a positive stain

Briefly, we used an Olympus IX81 light microscope to examine CD34-labelled immunohistological preparations. The microscope was controlled through a connected computer with the program, XCellence RT version 1.2 (Build 3554). Four nodal areas of each thyroid section were examined at 100× magnification, and one nodal area was examined at 25× magnification. It should be noted that we selected a central area that was representative of the node. Furthermore, the image acquisition settings were selected to ensure that the image sequences for all nodes had similar colour tones for the CD34-labelled areas. This similarity facilitated later analyses with the image processing software. Figure 3a shows two microscope images of thyroid gland sections immunohistochemically labelled with anti-CD34 at 100× and 25× magnifications.

With the image analysis program, CellProfiler version 3.1.5, we extracted two image sequences from the original image file; in this case, we extracted two images of the CD34-labelled immunohistological sections. The second image showed haematoxylin and PAS stains. The third image showed only the Fast-Red stained regions, which highlighted the CD34 antigen (Fig. 3b).

**Fig. 4:**
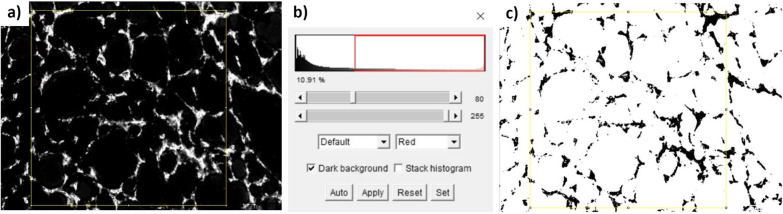
User interface of the image processing program, ImageJ, for evaluating CD34-labelled tissue sections. **a** An extracted image shows the fast-red-stained areas; a rectangular area (yellow box) is selected for analysis. **b** The threshold function renders the display to show **c** only image areas with a positive pixel value (black). Then, the measure key is used to evaluate the area of the CD34-positive portion (black portions of the right image) within the selection area (yellow rectangle), which is displayed in the Results window (bottom)

Finally, we used the image analysis program, ImageJ version 1.51J8, to examine the extracted Fast-Red image and determined the fraction of pixels that included Fast-Red stain among the total pixels in the ROI area. This fraction was taken as the quantitative measure of microscopically detectable vascularization (i.e., the fraction of vascularization). For this purpose, we selected a rectangular area within the image, the same size for all sections, with a pixel area of 2,077,920 (1440 × 1443 pixels), which corresponded to an actual area of 1 mm^2^. Figure 4 a-c shows an example of these steps. Next, the mean fraction of vascularization value was determined from four specific areas in the 100× magnified microscope image (called the *CD34 100 fold* fraction). Then, we selected a rectangular area of the same size within the 25× magnified image, and determined the *CD34 25 fold* fraction. Note that, for the 25× magnified image, this area corresponded to an actual area of 16 mm^2^ (1 mm^2^ × 4^2^). Finally, we averaged the mean vascularization fractions determined in the 100× and 25× magnified microscope images to obtain the overall average (the *CD34 all* fraction).

### Statistics

All statistical evaluations were performed with SAS Version 9.4. Results are expressed as the frequencies, mean values, and position and dispersion measures. Differences were determined using the non-parametric Wilcoxon rank sum test. The SMI results were available in quantitative form for evaluation. We applied the examination strategies described above to analyse quantitative characteristics. The relationships between quantitative parameters measured with the SMI approach and CD34-based histology were evaluated with the Spearman correlation. Potential confounding variables, such as age and body mass index, were taken into account with partial correlation analyses. *P*-values < 0.05 were considered statistically significant, according to the specified α = 0.05, with a probability of error of five percent.

## Results

### Patients

This study included 22 participants, and we examined 28 thyroid gland nodes sonographically. The statistical evaluations included 16 nodes of 16 patients. Of these, half of the participants were male. The average age was 39.75 years and the average body mass index was 26.08 kg/m^2^.

### Distribution of node proportions and dispositions

Among the 16 nodal lesions, 13 were benign (81.25%) and three were malignant (18.75%, Table 1). The malignant lesions included two papillary tumours (66.67%) and one follicular carcinoma (33.33%). The benign lesions included four benign neoplasms or follicular adenomas, four hyperplasias, and five undifferentiated lesions with benign findings. The nodes had an average length of 29.25 mm, with mean lengths of 27.92 mm in the benign group and 35 mm in the malignant group (*p* = 0.2807). The lesion volumes were also similar between groups (*p* = 0.6863).

**Table 1:** Distribution of patient characteristics and histological findings for 16 patients with thyroid gland nodes

Characteristic	Patients N (%)	Mean ± SD (range)
*Sex*		
Female Male	8 (50.00) 8 (50.00)	
*Body Mass Index*, kg/m^2^		
< 25 25–30 > 30	8 (50.00) 5 (31.25) 3 (18.75)	26.08 ± 5.18 (16.42–37.57)
*Age, years*		
< 18 18–30 31–40 > 40	0 (0.00) 4 (25.00) 6 (37.50) 6 (37.50)	39.75 ± 12.74 (24–68)
*Benign (n* = *13)*		
Benign neoplasia Benign hyperplasia Undifferentiated	4 (30.76) 4 (30.76) 5 (38.46)	
*Malignant (n* = *3)*		
Papillary Follicular Medullary Undifferentiated	2 (66.67) 1 (33.33) 0 (0.00) 0 (0.00)	

### Quantitative vascularization measurements, based on SMI and ImageJ

The vascularization parameters obtained with SMI were compared between malignant and benign thyroid lesions (Table 2). The mean (± SD) *Quotient longitudinal* values were similar between groups (0.88 ± 0.35 for benign nodes vs. 1.13 ± 0.46 for malignant nodes; *p* = 0.5012). The benign lesions and malignant tumours also had similar mean *Quotient longitudinal area fractions* (*p* = 0.2818), *Quotient alls* (*p* = 0.4273), and *Quotient all area fractions* (*p* = 0.2790).

**Table 2:** Differences between nodes with respect to vascularization parameters quantitatively determined with ImageJ and SMI

Variables	Benign (N = 13)		Malignant (N = 3)		*p*-value
	Mean ± SD	Median (range)	Mean ± SD	Median (range)	
ROI longitudinal	25.30 ± 10.69	22.33 (9.93–44.67)	26.55 ± 0.79	26.12 (26.07–27.46)	0.5905
ROI longitudinal area fraction (%)	0.25 ± 0.13	0.28 (0.02–0.43)	0.31 ± 0.04	0.30(0.27–0.35)	0.5905
ROI cross (mm^2^)	27.62 ± 11.13	23.68 (9.46–47.53)	24.11 ± 7.31	28.15 (15.68–28.51)	0.7879
ROI cross area fraction (%)	0.29 ± 0.15	0.27 (0.01–0.55)	0.23 ± 0.09	0.28 (0.12–0.28)	0.6865
Quotient longitudinal	0.88 ± 0.35	0.82 (0.37–1.67)	1.13 ± 0.46	1.18 (0.64–1.56)	0.5012
Quotient longitudinal area fraction	0.85 ± 0.66	0.64 (0.08–2.61)	2.75 ± 3.02	1.59 (0.48–6.17)	0.2818
Quotient all	0.88 ± 0.44	0.89 (0.28–1.54)	1.07 ± 0.05	1.07 (1.02–1.12)	0.4273
Quotient all area fraction	0.88 ± 0.89	0.58(0.14–3.28)	1.13 ± 0.19	1.21 (0.91–1.26)	0.2790

ROI: region of interest; longitudinal: longitudinal view of the ROI; area fraction: fraction of the area with blood flow compared to the total ROI area; cross: cross-sectional view of the ROI; Quotient: the indicated parameter measured in the thyroid gland lesion area (ROI-A) divided by the same parameter measured in the parenchymal tissue adjacent to the lesion (ROI-B); all: the quantified vascularization values measured in the total nodal area, divided by their counterparts measured in the parenchymal area, near the node in the same ROI

### Quantification of vascularization, based on CD34 labelling

In this study, the vascularization strength determined microscopically in CD34-labelled thyroid sections served as a reference, because it represented the actual blood flow strength in the thyroid node. The different vascularization parameters were compared between benign and malignant thyroid nodes (Table 3). These parameters were determined in tissue sections analysed with a light microscope at 25× and 100× magnifications. The mean vascularization fractions at 100 × magnification (*CD34 100 fold* fractions) were 0.05 for benign lesions and 0.08 for malignant differentiated nodes (*p* = 0.2260). The mean vascularization fractions at 25× magnification (*CD34 25 fold* fractions) were 0.04 and 0.06 for benign and malignant nodes, respectively (*p* = 0.4195). The average of the vascularization fractions measured at 100× and 25× magnifications (*CD34 all* fractions) were 0.04 and 0.07 for benign and malignant nodes, respectively (*p* = 0.4195).

**Table 3:** Differences between nodes in vascularization areas determined in 16 immunohistochemically stained CD34 sections

Variables	Benign (n = 13)		Malignant (n = 3)		*p*-value
	Mean ± SD	Median (range)	Mean ± SD	Median (range)	
CD34 100 fold area	0.05 ± 0.05	0.04 (0.01–0.20)	0.08 ± 0.06	0.07 (0.03–0.15)	0.2260
CD34 25 fold area	0.04 ± 0.04	0.02 (0.01–0.14)	0.06 ± 0.06	0.04 (0.02–0.12)	0.4195
CD34 all area	0.04 ± 0.04	0.03 (0.01–0.17)	0.07 ± 0.06	0.06 (0.02–0.14)	0.4195

CD34 100 fold area: the mean CD34-stained area measured in four specific rectangular regions selected in the 100× magnified microscope image; CD34 25 fold area: the CD34-stained area measured in a rectangular region selected in the 25× magnified image; CD34 all area: The average of the mean CD34-stained areas measured in the 100× and 25× magnified microscope images

### Relationship between CD34 expression and quantitatively determined SMI vascularization

Finally, we investigated whether the CD34 expression evaluated in the immunohistological preparations were correlated with the vascularization strength evaluated with SMI imaging and ImageJ analysis. The Spearman correlation coefficients indicated the correlation strength between vascularization parameters determined from SMI and CD34 measurements (Table 4). We found no significant correlation between the *Quotient longitudinal* and the *CD34 100 fold*, the *CD34 25 fold*, or the *CD34 all* (*p* = 0.40588, 0.49118, and 0.43235, respectively). However, the vascularization parameter *Quotient longitudinal area fraction* was significantly correlated with all three variables derived from CD34 staining (*p* = 0.0254, 0.0374, and 0.0350, respectively). For some vascularization parameters, such as *Quotient all*, we observed partially negative correlations.

**Table 4:** Spearman correlations between vascularization areas determined from CD34-stained images and those determined from SMI measurements

Parameters	Spearman correlation coefficient (r) *p*-values			
	CD34 100 fold		CD34 25 fold	CD34 all
ROI longitudinal	0.14706		0.05882	0.07353
	0.5868		0.8287	0.7867
ROI longitudinal area fraction (%)	0.10882		− 0.00294	0.03824
	0.6883		0.9914	0.8882
ROI cross	− 0.11765		− 0.34118	− 0.28824
	0.6643		0.1959	0.2790
ROI cross area fraction (%)	− 0.34412		− 0.39412	− 0.38824
	0.1919		0.1309	0.1373
Quotient longitudinal	0.40588		0.49118	0.43235
	0.1188		0.0534	0.0944
Quotient longitudinal area fraction (%)	0.55588		0.52353	0.52941
	0.0254		0.0374	0.0350
Quotient cross	0.07941		0.06765	0.03824
	0.7700		0.8034	0.8882
Quotient cross area fraction (%)	0.06786		− 0.06071	− 0.01429
	0.8101		0.8298	0.9597
Quotient all	0.11429		− 0.02857	− 0.06071
	0.6851		0.9195	0.8298
Quotient all area fraction (%)	0.08571		− 0.03214	− 0.07500
	0.7613		0.095	0.7905

CD34 100 fold: the mean CD34-stained area measured in four specific rectangular regions selected in the 100× magnified microscope image; CD34 25 fold area: the CD34-stained area measured in a rectangular region selected in the 25× magnified image; CD34 all area: The average of the mean CD34-stained areas measured in the 100×  and 25× magnified microscope images; ROI: region of interest; longitudinal: longitudinal view of the ROI; area fraction: fraction of the area with blood flow compared to the total ROI area; cross: cross-sectional view of the ROI; Quotient: the indicated parameter measured in the thyroid gland lesion area (ROI-A) divided by the same parameter measured in the parenchymal tissue adjacent to the lesion (ROI-B); all: the quantified vascularization values measured in the nodal area of ROI-total, divided by their counterparts measured in the parenchymal area, near the node in the same ROI

**Fig. 5:**
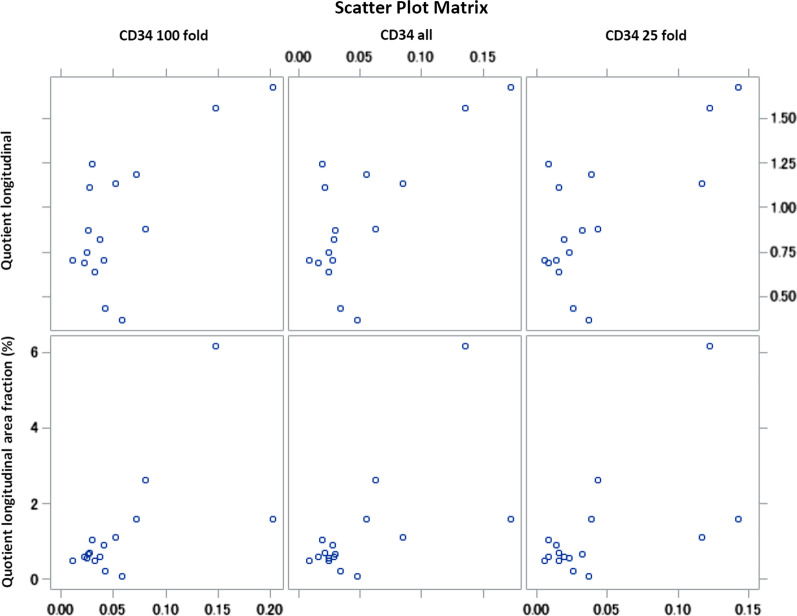
Residual scatter plots for analysing correlations between vascularization parameters. (Top) The *Quotient longitudinal* and (bottom) the *Quotient longitudinal area fraction* are plotted against the vascularization variables, (left) *CD34 100 fold*, (centre) *CD34 all,* and (right) *CD34 25 fold* (also see Table 4)

Figure 5 shows the correlations between the SMI variables, *Quotient longitudinal* and *Quotient longitudinal area fraction*, and the CD34 parameters, *CD34 100 fold*, *CD34 25 fold*, and *CD34 all*, in the form of point clouds.

## Discussion

### Sonographic evaluation of vascularization with ImageJ

This is the first study to quantify the vascularization of thyroid nodules detected by SMI using computer analysis by ImageJ. Our results showed that there was no significant difference between benign and malignant nodes, based on the SMI vascularization parameter.

Previous studies have only undertaken the quantitative translation of thyroid nodules in colour Doppler or Power Doppler imaging mode [26, 35, 36]. Wu et al. applied Power Doppler imaging and the program, AmCAD-UV, to thyroid nodes, and they also determined a vascular index, which they correlated with node disposition [36].^.^In their study, the vascularization in the node was determined over a period of time. However, Wu et al. showed that malignant nodes had significantly lower vascularization values than benign lesions. That finding was directly opposed to the assumption that malignant tumours are characterized by stronger vascularization than benign lesions.

In summary, it can be said that we quantified the vascularization in thyroid nodes with a method that had been successfully used in several previous studies on other organ systems [26, 33, 36]. However, we found no significant difference between malignant and benign nodes, in terms of our vascularization parameters. One way to provide more accurate or objective measurements might be to use, like Wu et al., several frames of a few seconds of blood flow for the software analysis. However, in that study, the results partly contradicted the current state of the study the assumption, that malignant lesions show increased vascularization. Therefore, it is doubtful that vascularization alone could serve as a criterion for evaluating malignancy.

### Quantified CD34 expression

In this study, we concluded that CD34 expression was not significantly different between malignant and benign thyroid lesions. The marginally higher incidence of CD34 positivity that we observed in malignant nodules compared to benign lesions contradicted previous results from comparable investigations, such as the study by Jiang et al. In that study, malignant tumours showed less CD34 expression than benign lesions [37]. This discrepancy might be explained, in part, by the fact that, our collection, unlike their collection, included follicular carcinomas, which are known to be more highly vascularized than other tumours. Furthermore, this discrepancy might also be explained, in part, by the fact that they used manual counting as an alternative method for quantitative determinations of CD34 expression in the node. Finally, the explanation for the discrepancy might also include their closer differentiation of CD34-labelled areas, based on sorting by vessel diameters to facilitate the selection of microvessels, as performed by Sancak et al. [38]. In contrast, in the present study, we investigated the histological correlate for blood flow, which was generally independent of vessel size, to facilitate a comparison to the blood flow determined with SMI. Moreover, this Doppler method did not differentiate according to vessel diameter. Although SMI can represent microvessels better than other methods, it also included larger vessels, which contributed substantially to the quantitatively determined vascularization strength. In addition, it should be noted that some previous studies have shown that the expression of vascular factors had only low prognostic value [27, 39, 40].

### Correlation between CD34 und ImageJ

Finally, we investigated the question of whether the vascularization measure determined quantitatively with SMI and ImageJ could coherently reflect the actual vascularization, which was determined with CD34 staining and computer image evaluations. Our Spearman correlation coefficients indicated that the most promising results were achieved with the parameters, *Quotient longitudinal area fraction* and *CD34 100 fold.* Nevertheless, the significance of this result must be questioned. Other vascularization parameters, such as the *Quotient cross area fraction* and the *Quotient all*, showed negative correlations to the vascularization variables determined with CD34. These results were interpreted as contradictory. Consequently, we must consider the possibility that the significant results might have occurred coincidentally.

Only a few previous studies investigated the relationship between sonographically and immunohistologically determined vascularization in thyroid nodes [39, 41, 42]. However none of these studies provided a comparison between quantified parameters representing the vascularization, derived from SMI Doppler imaging on the one hand and immunshistologically analyzation on the other hand. In contrast, the present study placed emphasis on the use of image processing software to ensure the highest possible objectivity.

It might be possible to achieve better results by selecting the smallest possible ROI and evaluating exclusively the central area of the thyroid node in SMI mode. This approach might provide better results, because a small Doppler sonogram window of 5 × 5 mm would be more comparable to the histological areas of 1 mm^2^ and 16 mm^2^, which were evaluated under the microscope at 100× and 25× magnifications, respectively. In addition, the area with the strongest blood supply ("hot spots") should be selected in the overall image of the node, because this selection was shown to be advantageous by Wong et al. [39].

Some previous studies have shown that an increased microvascular density should be a malignancy criterion, rather than hypervascularization, per se [38]. Therefore, it might be advantageous to differentiate accordingly when recording the blood circulation properties of a node. In histological analyses, it is possible to include vessels up to a certain size in the evaluation. This type of categorization is difficult to achieve in sonographic data. However, SMI has the particular strength of detecting microvessels better than other Doppler methods; thus, small calibre vessels should make more of an impact on the SMI blood flow images.

In summary, we could not show a correlation between histological and SMI vascularization parameters with certainty. A comparison with other studies was limited, because no previous study has investigated this correlation with SMI as the Doppler sonographic method.

This work is a pilot study which had the aim to test the study design and its realizability. However, this study has certain limitations. First, only a small number of patients was included in our research, which took place at a single center. The very small sample size must be considered as a major limitation. Small effects and differences between the groups can only be determined with difficulty and in a limited way with the non-parametric methods. In order to gain more accurate and representative statistical results the research should be extended to a larger cohort, for example by extension to a multicenter-study. Second, acquisition of ultrasound examination was performed by only one investigator. To ensure better objectivity, the execution of ultrasound examination should be done by a certain number of experts to avoid interobserver variability, at least two experts should record the sonographic characteristic. If disagreement came up, a third expert should make the final determination. Third, only patients were included in this study who were planned for surgery. Either they had symptomatic thyroid nodes or they were suspicious for having thyroid cancer. By this selection cases with malignant nodes might be overrepresented.

## Conclusion

However, for blood flow sonography, it would be desirable to capture microvasculature to the exclusion of larger vessels. Based on previous histological studies, only the microvascular contribution to vascularization strength appears to be a decisive criterion for the differentiation between benign lesions and malignant tumours. Based on our results, further studies on larger collectives seem to be indicated to map small effects and to assess clinical applicabilitiy and utility.

## Data Availability

The datasets generated and analysed during the current study are not publicly available but are available from the corresponding author on reasonable request.

## Abbreviations

ROI: Region of interest
SMI: Superb microvascular imaging
